# Psoriasis and dementia: a population‐based matched cohort study of adults in England

**DOI:** 10.1002/alz.086089

**Published:** 2025-01-09

**Authors:** Julian Matthewman, Sharon L Cadogan, Katrina Abuabara, Krishnan Bhaskaran, Catherine Smith, Kathryn E Mansfield, Sinéad Langan, Charlotte Warren‐Gash

**Affiliations:** ^1^ London School of Hygiene and Tropical Medicine, London, London United Kingdom; ^2^ London School of Hygiene and Tropical Medicine, London United Kingdom; ^3^ UCSF, San Francisco, CA USA; ^4^ London School Of Hygiene And Tropical Medicine, London United Kingdom; ^5^ Kings College London, London United Kingdom

## Abstract

**Background:**

Substantial evidence suggests association between increased inflammatory markers and Alzheimer’s disease. However, evidence for association between the inflammatory skin disease psoriasis and dementia is limited and conflicting. Additionally, few studies investigate how psoriasis severity influences risk.

**Method:**

We used primary care electronic health records from the UK’s Clinical Practice Research Datalink Aurum and linked hospital admissions data for a matched cohort study (1997‐2022) of individuals ≥40years. We matched (age, sex, primary care practice) individuals with psoriasis, without replacement, with up to five comparators without psoriasis in calendar date order. People with psoriasis were followed from latest of: first psoriasis diagnosis, practice registration +1year, study start, age 40, date practice met quality‐control standards. Comparators were followed from the same date as their psoriasis match. We used Cox regression, stratified by matched set, to estimate hazard ratios (HR) comparing dementia risk in those with and without psoriasis.

**Result:**

In preliminary analyses, we identified 360,014 individuals with psoriasis matched to 1,799,617 without. After implicitly adjusting for age, sex, practice and calendar period (through underlying timescale and matching), and adjusting for lifestyle factors (smoking, harmful alcohol, obesity) and comorbidities (cardiovascular, cerebrovascular, chronic liver and chronic lung diseases, diabetes mellitus, depression, hyperlipidaemia, hypertension), we found evidence of association between psoriasis and all‐cause dementia (HR 1.04, 95%CI 1.01‐1.06), although absolute rate differences were small (approximately 4 per 10,000 person years). We found evidence of association between psoriasis and vascular dementia (HR 1.09, 95%CI 1.04‐1.15), but no evidence of association with Alzheimer’s (HR 1.02, 95%CI 0.98‐1.05). Compared to those without psoriasis, the association with all cause dementia was stronger in moderate‐to‐severe psoriasis (HR 1.17, 95%CI 1.07, 1.27) than in mild psoriasis (HR 1.02, 95%CI 1.00, 1.04).

We plan to: further adjust for additional potential confounders (including deprivation, ethnicity, chronic kidney disease) using more nuanced sequential modelling; and assess how robust our findings are in sensitivity analyses.

**Conclusion:**

Our findings suggest that risk of all‐cause dementia, but not Alzheimer’s, is slightly higher in people with psoriasis, and risk is greater in more severe psoriasis. However, relative risk and absolute risk differences were small.

